# Prognostic Impact of Muscle Quantity and Quality and Fat Distribution in Diffuse Large B-Cell Lymphoma Patients

**DOI:** 10.3389/fnut.2021.620696

**Published:** 2021-05-07

**Authors:** Giulia Besutti, Fulvio Massaro, Efrem Bonelli, Luca Braglia, Massimiliano Casali, Annibale Versari, Guido Ligabue, Pierpaolo Pattacini, Silvio Cavuto, Domenico F. Merlo, Stefano Luminari, Francesco Merli, Salvatore Vaccaro, Massimo Pellegrini

**Affiliations:** ^1^Radiology Unit, Department of Imaging and Laboratory Medicine, Azienda Unità Sanitaria Locale – IRCCS di Reggio Emilia, Reggio Emilia, Italy; ^2^Clinical and Experimental Medicine PhD Program, University of Modena and Reggio Emilia, Modena, Italy; ^3^Hematology Unit, Azienda Unità Sanitaria Locale – IRCCS di Reggio Emilia, Reggio Emilia, Italy; ^4^Research and Biostatistics Unit, Azienda Unità Sanitaria Locale – IRCCS di Reggio Emilia, Reggio Emilia, Italy; ^5^Nuclear Medicine Unit, Oncology Department, Azienda Unità Sanitaria Locale – IRCCS di Reggio Emilia, Reggio Emilia, Italy; ^6^Radiology Unit, Azienda Ospedaliero-Universitaria Policlinico di Modena, Modena, Italy; ^7^Clinical Nutrition Unit, Azienda Unità Sanitaria Locale – IRCCS di Reggio Emilia, Reggio Emilia, Italy; ^8^Department of Biomedical, Metabolic and Neural Sciences, University of Modena and Reggio Emilia, Modena, Italy

**Keywords:** sarcopenia, diffuse large b-cell lymphoma, computed tomography, body composition, progression-free survival, overall survival

## Abstract

Baseline CT scans of 116 patients (48% female, median 64 years) with diffuse large B-cell lymphoma (DLBCL) were retrospectively reviewed to investigate the prognostic role of sarcopenia and fat compartment distributions on overall survival (OS), progression-free survival (PFS), and early therapy termination. Skeletal muscle index (SMI), skeletal muscle density (SMD), and intermuscular adipose tissue (IMAT) were quantified at the level of the third lumbar vertebra (L3) and proximal thigh (PT). Low L3-SMD, but not low L3-SMI, was associated with early therapy termination (*p* = 0.028), shorter OS (HR = 6.29; 95% CI = 2.17–18.26; *p* < 0.001), and shorter PFS (HR = 2.42; 95% CI = 1.26–4.65; *p* = 0.008). After correction for sex, International Prognostic Index (IPI), BMI, and R-CHOP therapy (rituximab, cyclophosphamide, doxorubicin, vincristine, prednisone), low L3-SMD remained associated with poor OS (HR = 3.54; 95% CI = 1.10–11.40; *p* = 0.034) but not with PFS. Increased PT-IMAT was prognostic for poor OS and PFS after correction for sex, IPI, BMI, and R-CHOP therapy (HR = 1.35; CI = 1.03–1.7; *p* = 0.03, and HR = 1.30; CI = 1.04–1.64; *p* = 0.024, respectively). Reduced muscle quality (SMD) and increased intermuscular fat (IMAT), rather than low muscle quantity (SMI), are associated with poor prognosis in DLBCL, when measured at the L3 level, and particularly at the level of the proximal thigh. The proximal thigh represents a novel radiological landmark to study body composition.

## Introduction

Non-Hodgkin lymphoma is the fifth most common neoplasia in Western countries (1). Among the various histological subtypes, DLBCL accounts for 25–35% of all cases (2). With the advent of modern chemoimmunotherapy regimens, more than 50% of DLBCL patients achieve complete remission (3). Nevertheless, many patients still fail to achieve an optimal response or experience relapse, and the most used prognostic tools to identify these subjects comprise only few clinical features (age, stage, performance status, serum lactate dehydrogenase level, number of involved extranodal sites) that were validated more than 20 years ago (4).

Body composition parameters are associated with long-term health outcomes in many diseases and have been recently studied as prognostic factors in DLBCL patients. Researchers have reported a negative impact of sarcopenia on survival outcomes in DLBCL patients treated with the R-CHOP regimen (5–7), particularly among elderly patients (5).

However, a conclusive definition of sarcopenia has not yet been established, and its assessment often remains beyond clinical practice. A CT scan is one of the most widely accepted tools for the assessment of sarcopenia, defined as severe systemic loss of skeletal muscle mass leading to progressive functional impairment. CT or PET-CT scan is routinely performed in DLBCL patients during initial staging and subsequent follow-up, representing an accessible source of data for the identification of sarcopenia or abnormal fat accumulation.

Sarcopenia, defined as a decreased skeletal muscle index at the level of the third lumbar vertebra, yielded ambiguous results as a prognostic marker. Some authors found that a decreased muscle mass was independently associated with lower overall survival and progression-free survival in DLBCL patients (5, 7–9). Others confirmed this result in male patients only (10, 11), while Chu et al. (6, 12) described a trend toward an improved survival in patients with lower SMI.

Besides muscle depletion, sarcopenia is also characterized by an increased proportion of inter- and intramuscular fat, which are markers of muscle quality deterioration (13, 14). Skeletal muscle density, evaluated by the mean attenuation on CT imaging, represents the accumulation of intramuscular fat and water, while intermuscular adipose tissue is the quantity of visible fat that can be measured beneath the fascia and within the muscles. Both SMD and IMAT are predictors of muscle function (15, 16). Furthermore, these ectopic fat depots are associated with chronic inflammation, metabolic impairment, and insulin resistance (17). A recent meta-analysis reported the association between low skeletal muscle density and prognosis in cancer patients (18). As described for non-hematologic malignancies (19, 20), a reduced SMD is a better predictor of poor survival than a reduced SMI in DLBCL patients (6, 12).

During cancer trajectory, inflammation, metabolic derangements, poor nutrition, physical inactivity, and cancer treatment may result in simultaneous skeletal muscle loss and fat gain that culminate in sarcopenic obesity, which is strongly related to reduced survival in cancer patients (21). Several studies have addressed the association between body mass index (BMI) and DLBCL prognosis with discordant results (22–25), while a higher visceral adiposity measured by means of a CT scan resulted in a poorer prognosis (26). Recently, the combination of sarcopenia and adipopenia in DLBCL patients has been associated with patients' outcomes (10), while in lymphoma patients undergoing stem cell transplantation, the combined effect of visceral adiposity and sarcopenia defined as low SMI has been correlated with higher mortality (27). However, the potential combined prognostic effect of low muscle quality and visceral adiposity has never been investigated in DLBCL patients.

The aim of this study was to explore the impact of baseline PET-CT indices of sarcopenia and adipose tissue distribution on OS and PFS in patients with DLBCL. Secondarily, we aimed to evaluate the impact of these indices on early therapy termination.

We have explored body composition metrics in PET-CT cross-sectional images at the L3 level and at the proximal thigh (PT) level. While the L3 cross-sectional skeletal muscle area (SMA) is a standard landmark linearly related to whole-body muscle mass and total adipose tissue (28), the skeletal muscle cross-sectional area at the PT level might be a new and sensitive prognostic marker of sarcopenia.

## Materials and Methods

### Patients and Study Design

All consecutive patients diagnosed with DLBCL between January 2014 and December 2017 at our institution were considered eligible for inclusion in this retrospective study. All patients were diagnosed through lymph node excision biopsy or core biopsy of the presenting extranodal sites and underwent a unilateral bone marrow biopsy. DLBCL patients routinely undergo whole-body PET-CT and CT imaging at the time of diagnosis for disease staging. The unavailability of baseline PET-CT scan for retrospective review was considered as an exclusion criterion. Patients informed consent to participate in the study was obtained whenever possible, due to the retrospective nature of the study. The study was approved by the appropriate local ethics committee of Area Vasta Emilia Nord (protocol number: 2018/0111083) and have therefore been performed in accordance with the ethical standards laid down in the 1964 Declaration of Helsinki and its later amendments.

### Clinical Chart

Patient records were retrospectively reviewed to collect demographic data (age, sex), anthropometric data at the time of diagnosis (height, weight, and BMI, defining obesity as a BMI ≥30), and baseline known clinical prognostic factors [Ann Arbor disease staging, serum lactate dehydrogenase level, Eastern Cooperative Oncology Group (ECOG) performance status, extranodal sites]. The International Prognostic Index (IPI) summarizing all of the aforementioned prognostic factors was calculated for all patients. Data on chemoimmunotherapy regimen, drug-induced toxicity, and response to therapy were collected. After evaluation of disease features, age, and comorbidities, the patients received chemoimmunotherapy treatment with different regimens according to international guidelines and local practice.

### PET-CT Image Analysis

PET data were acquired using a Discovery GE PET/CT scanner (General Electric Medical Systems, Cleveland, OH, USA), which combines a helical 16-slice CT and a 3-dimensional (3-D) PET scanner. Body composition parameters were measured on the non-enhanced CT helical scan (scan field 500 mm, increment 3.75 mm, slice thickness 3.75 mm, pitch 1. 0.8 s per rotation, matrix 512 × 512 pixels, 120 kV, 80 mA). Images were retrospectively analyzed by a single trained image analyzer (EB) supervised by a senior radiologist (PP), both blinded to clinical data and outcomes, using the OSIRIX-Lite software V5.0 (Pixmeo, Sarl, Switzerland).

**Figure 1:**
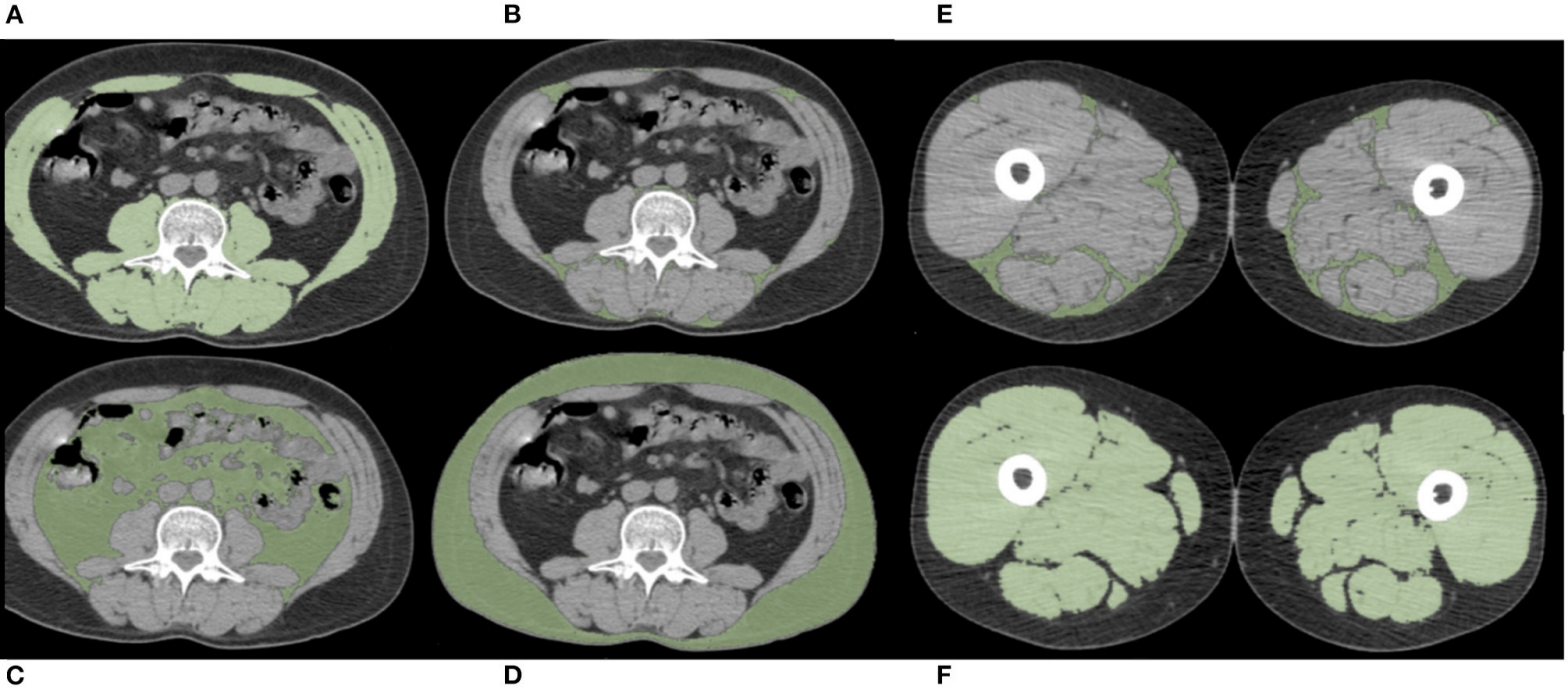
Examples of PET-CT body composition measurements. Muscle and adipose tissue segmentations were obtained after applying specific density thresholds. In these axial slices at the level of the third lumbar vertebra (L3) **(A–D)** and at the level of proximal thigh (PT) **(E,F)**, the colored regions of interest represent L3- and PT-skeletal muscle area (SMA) **(A,F)**, L3- and PT-intermuscular adipose tissue (IMAT) **(B,E)**, visceral adipose tissue (VAT) **(C)**, and subcutaneous adipose tissue (SAT) **(D)**.

A single slice at the L3 level with both transverse processes visible was selected. Skeletal muscle and abdominal fat compartments were selected after applying Hounsfield Unit (HU) thresholds of −29 to +150 and −190 to −30, respectively. The cross-sectional SMA and the total adipose tissue, subcutaneous adipose tissue (SAT), visceral adipose tissue (VAT), and IMAT at the L3 level were obtained through autosegmentation and manual contour correction when necessary. The muscles segmented included the rectus abdominis, abdominal wall, psoas, and paraspinal muscle groups. The SMI was calculated by dividing the cross-sectional SMA by squared height in meters. Decreased muscle quantity was defined according to previously used L3-SMI cut-off values (<43 cm^2^/m^2^ for men with BMI <25, <53 cm^2^/m^2^ for men with BMI ≥25, and <41 cm^2^/m^2^ for women) (29). Other previously used cut-off values were also investigated: L3-SMI-2 (<55.8 cm^2^/m^2^ for men and <38.9 cm^2^/m^2^ for women) (5) and L3-SMI-3 (<52.4 cm^2^/m^2^ for men and <38.5 cm^2^/m^2^ for women) (30). Unless otherwise specified, the analysis of L3-SMI using the cut-off value of Martin et al. (29) is reported. The analysis using other cut-off parameters (L3-SMI-2 and L3-SMI-3) is reported in the Electronic Supporting Information. The Mean SMD (L3-SMD) was registered in the same region of interest used for SMA measurement. Poor skeletal muscle quality was defined according to previously used L3-SMD cut-off values (<41 HU for patients with BMI <25 and <33 HU for patients with BMI >25) (10, 29, 30). VAT/SAT ratio was also calculated to describe the relative distribution of adipose tissue in body fat compartments. Using the same software and analytical method, IMAT, SMA, SMI, and SMD were also obtained at the PT level. The CT slice selected for this purpose was immediately below the last slice including the gluteal muscle. Representative images are reported in Figure 1.

### Statistical Analyses

In the absence of a priori hypothesis, given the exploratory nature of the study, no formal sample size calculation was performed. Clinical and demographic data were expressed in terms of frequency and percentage for categorical variables, mean ± standard deviation for approximately normally distributed quantitative variables, and median [and interquartile range (IQR)] otherwise. Proportion estimates were accompanied by Wilson (31) confidence intervals (CIs). Proportions were compared between independent groups using the chi-square test or Fisher's exact test if needed. Quantitative variables were compared across independent groups using the Mann-Whitney U test; correlation was assessed with the Pearson correlation coefficient (after visual inspection of scatterplots).

In terms of prognostic analyses, PFS was defined as the time from the diagnosis to first progression or death from any cause, whichever came first. OS was defined as the time from the diagnosis to death from any cause. The median follow-up time was evaluated using the reverse Kaplan-Meier method (32). Prognostic analyses entailed both estimates of survival function (using the Kaplan-Meier method, compared using the log rank test) and estimates of effects of covariates via Cox's regression models (proportional hazard assumption was assessed by testing scaled Schoenfeld residuals' correlation with time; no violation of the assumption was found).

Main prognostic analyses included PET-CT indices, both continuous and dichotomized, according to the criteria proposed in the literature; in multivariate analyses, we used IPI, sex, BMI, and therapy as covariates. Unless otherwise specified, CIs for estimates were two-tailed and calculated considering a 0.95 confidence level. Performed tests were considered statistically significant for *p*-values <0.05. Statistical analysis was performed using R 3.5.2 (2020) (R Core Team, Vienna, Austria).

## Results

### Population

Of 120 patients diagnosed with DLBCL between January 2014 and December 2017 at our institution, 4 were excluded because of unavailability of PET-CT examination performed at baseline for staging purposes, leaving a total of 116 patients included in the study. The mean age at diagnosis was 63.7 (±16.4) years and 56 patients (48.3%) were female. Demographic, anthropometric, and clinical characteristics at the time of diagnosis and data on therapy and response to therapy are reported in Table 1.

**Table 1:** Demographic, anthropometric, and clinical data of the patients at the time of diagnosis and chemoimmunotherapy regimen.

**Covariates**		**All patients (*n* = 116)**
Age at diagnosis (years)		63.7 ± 16.4
Female		56 (48.3%)
Weight (kg)		70.6 ± 15.1
Height (cm)		166.0 ± 10
BMI (kg/m^2^)		25.4 ± 4.4
Stage	I	14 (12.1%)
	II	24 (20.7%)
	III	17 (14.6%)
	IV	61 (52.6%)
ECOG performance status	0	80 (69.0%)
	1	12 (10.3%)
	2	24 (20.7%)
Extranodal sites	0	37 (32.2%)
	1	45 (39.1%)
	2	25 (21.7%)
	3	6 (5.2%)
	4	2 (1.7%)
LDH (U/l)		468 (338–656)
IPI	0	8 (6.9%)
	1	24 (20.7%)
	2	24(20.7%)
	3	30 (25.9%)
	4	23 (19.8%)
	5	7 (6%)
Chemo-immunotherapy regimens	R-CHOP	70 (60.3%)
	Others^a^	46 (39.7%)
Early therapy termination		16 (13.8%)
Response to therapy	Not evaluated	9 (7.8%)
	Progressive disease	6 (5.2%)
	Complete remission	86 (74.1%)
	Partial remission	15 (12.9%)

*Data are reported as frequencies and percentage for categorical variables, means and standard deviations for normally distributed continuous variables, and median and interquartile range for non-normally distributed continuous variables. BMI, body mass index; ECOG, Eastern Cooperative Oncology Group; IPI, international prognostic index*.

a *Includes chemoimmunotherapy regimens R-CVP (n = 5), R-miniCHOP (n = 18), R-MACOP-B (n = 9), dose-adjusted EPOCH-R (n = 3), R-CODOX-M/R-IVAC (n = 3), autologous stem cell transplantation with FEAM conditioning (n = 8) (33–35). R-CHOP, rituximab, cyclophosphamide, doxorubicin, vincristine, prednisone; R-CVP, rituximab, cyclophosphamide, vincristine, prednisone; R-miniCHOP, rituximab, cyclophosphamide, doxorubicin, vincristine, prednisone; R-MACOP-B, rituximab, methotrexate, doxorubicin, cyclophosphamide, vincristine, prednisone, bleomycin; Dose-adjusted EPOCH-R, etoposide, vincristine, doxorubicin, cyclophosphamide, prednisone, rituximab; R-CODOX-M/R-IVAC, rituximab, cyclophosphamide, vincristine, doxorubicin, methotrexate / rituximab, ifosfamide, etoposide, cytarabine; FEAM, fotemustine, etoposide, cytarabine, melphalan*.

### PET-CT Indices

Body composition PET-CT indices at the time of diagnosis are reported in Supplementary Table 1. The prevalence of L3-SMI-defined decreased muscle mass was 25%, while the prevalence of poor muscle quality based on L3-SMD was 52.6%. In terms of the associations between baseline PET-CT indices and serologic data such as glycemia, albumin, total serum protein, C-reactive protein, and vitamin D, we found correlations between L3-SMD and albumin (*r* = 0.38, CI: 0.22–0.53), L3-SMD and total serum protein level (*r* = 0.41, CI: 0.24–0.55), and VAT and glycemia (*r* = 0.35, CI: 0.18–0.50). Upon examining the associations between different PET-CT indices, we identified a weak correlation between L3-SMD and L3-SMI (*r* = 0.36, CI: 0.19–0.51). Muscle quantity and quality indices (SMD, SMI, and IMAT) measured at the L3 level and PT level showed strong correlations, with r ranging between 0.63and 0.86, the lowest for IMAT and the highest for SMD, respectively. These results are reported in Supplementary Table 2.

### Outcome Measures

The median follow-up was 30 months (IQR, 24–34 months). During the follow-up, we observed 43 progressions, of which 28 were deaths. The median OS and PFS were 64.8 and 53.6 months, respectively. Survival curves for the whole study population are reported in Supplementary Figure 1.

### Impact of Body Composition PET-CT Indices on Survival

Considering PET-CT indices as continuous variables (Table 2), an increased SMD was found to be a protective factor both for OS and PFS, which was stronger at the PT level, with significant or borderline significant *p*-values in the multivariate analysis after correction for sex, BMI, IPI, and therapy (HR = 0.46; CI = 0.23–0.90; *p* = 0.025, and HR = 0.62; CI = 0.37–1.04; *p* = 0.068, respectively). The SMI showed discordant effects; however, an increased L3-SMI was a significant independent poor prognostic factor for OS in the multivariate analysis (HR = 2.06; CI = 1.11–3.83; *p* = 0.023). An increased IMAT had a significant independent poor prognostic role both in OS and PFS when measured at the PT level (HR = 1.35; CI = 1.03–1.7; *p* = 0.03, and HR = 1.30; CI = 1.04–1.64; *p* = 0.024, respectively), but not at the L3 level. Other indices including VAT and VAT/SAT did not show any significant association with neither OS nor PFS.

**Table 2:** Univariate and multivariate associations of different PET-CT indices with OS and PFS.

	**Univariate analysis**			**Multivariate analysis**		
	**HR**	**95% CI**	**p**	**HR**	**95% CI**	***p***
**Overall survival**						
L3-SMI	1.24	0.86–1.79	0.240	2.06	1.11–3.83	0.023
L3-SMD	0.46	0.29–0.74	0.001	0.59	0.33–1.05	0.075
L3-IMAT	1.22	0.89–1.68	0.211	0.94	0.65–1.36	0.742
PT-SMI	0.85	0.72–1.01	0.069	1.02	0.82–1.27	0.872
PT-SMD	0.28	0.16–0.50	<0.001	0.46	0.23–0.90	0.025
PT-IMAT	1.41	1.12–1.78	0.003	1.35	1.03–1.76	0.030
VAT	1.01	0.97–1.04	0.795	1.01	0.95–1.07	0.799
VAT/SAT	1.14	0.71–1.83	0.596	0.87	0.47–1.62	0.658
**Progression free survival**						
L3-SMI	0.96	0.69–1.33	0.797	1.16	0.71–1.90	0.561
L3-SMD	0.64	0.44–0.91	0.014	0.83	0.55–1.26	0.387
L3 IMAT	1.09	0.85–1.41	0.487	0.89	0.67–1.18	0.405
PT-SMI	0.86	0.74–0.98	0.028	1.00	0.83–1.19	0.982
PT-SMD	0.41	0.26–0.63	<0.001	0.62	0.37–1.04	0.068
PT-IMAT	1.33	1.09–1.62	0.004	1.30	1.04–1.64	0.024
VAT	1.01	0.98–1.04	0.584	1.03	0.98–1.08	0.197
VAT/SAT	1.26	0.89–1.80	0.192	1.12	0.73–1.72	0.595

*The multivariate models include IPI, sex, BMI, and therapy (R-CHOP vs. other regimens) as covariates. PET-CT, positron emission tomography-computed tomography; OS, overall survival; PFS, progression-free survival; IPI, international prognostic index; BMI, body mass index; HR, hazard ratio; CI, confidence interval; L3, third lumbar vertebra; PT, proximal thigh; SMI, skeletal muscle index; SMD, skeletal muscle density; IMAT, intramuscular adipose tissue; VAT, visceral adipose tissue; SAT, subcutaneous adipose tissue. HRs for SMI, SMD, IMAT, and VAT are reported considering a 10-unit increase; R-CHOP, rituximab, cyclophosphamide, doxorubicin, vincristine, prednisone; R-CVP, rituximab, cyclophosphamide, vincristine, prednisone; for other regimens see Table 1*.

### Effect of Poor Muscle Quality and Muscle Mass Depletion on Survival

**Figure 2:**
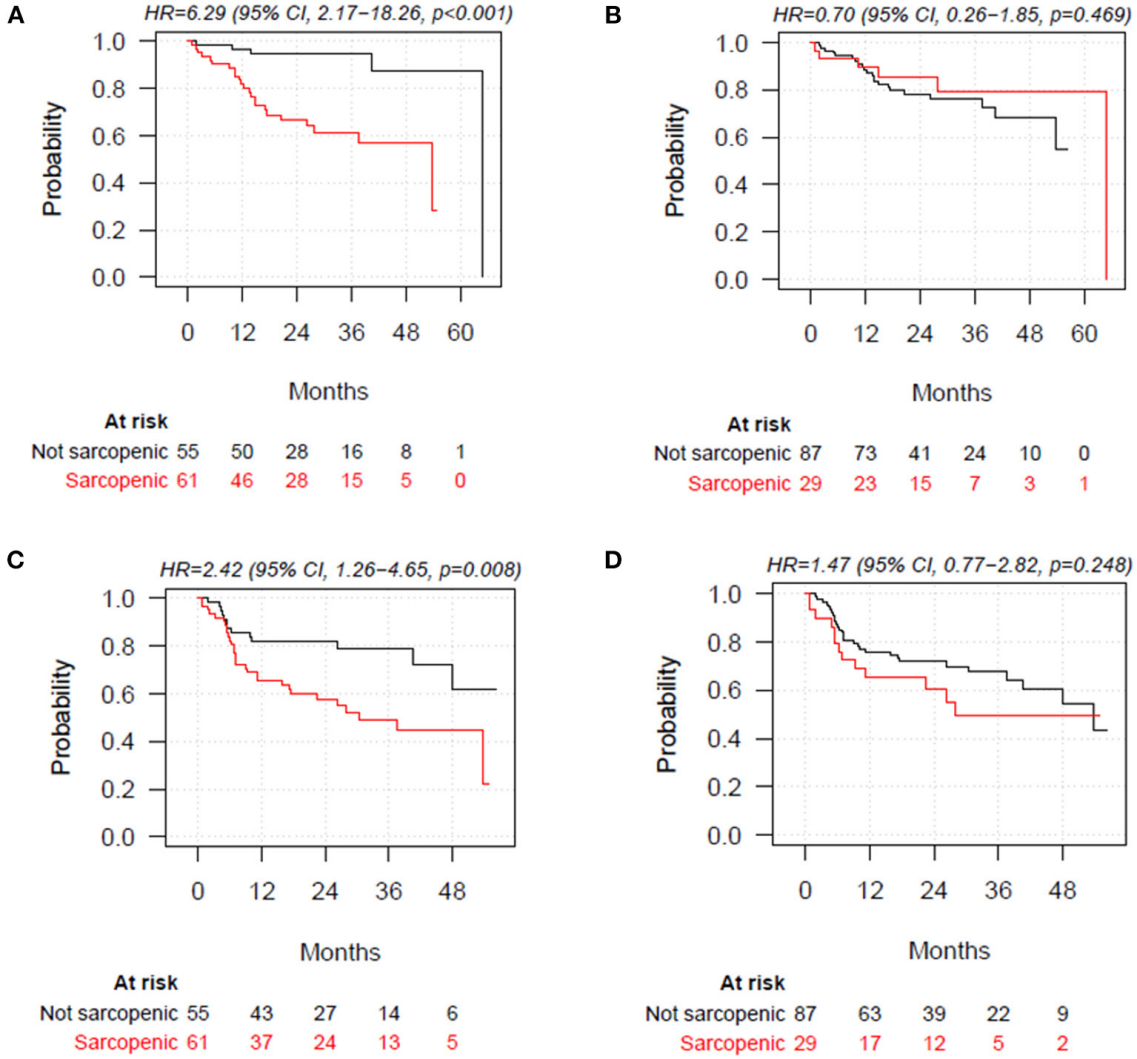
Overall survival stratified by poor muscle quality defined according to skeletal muscle density at the level of the third lumbar vertebra (L3-SMD), **(A)** and decreased muscle mass according to skeletal muscle index at the level of the third lumbar vertebra (L3-SMI), (cut-off values: <43 cm^2^/m^2^ for men with BMI <25, <53 cm^2^/m^2^ for men with BMI ≥25, and BMI<41 cm^2^/m^2^ for women) **(B)**. Progression-free survival (PFS) stratified by poor muscle quality defined according to L3-SMD (<41 HU for patients with BMI <25 and <33 HU for patients with BMI >25) **(C)** and decreased muscle mass according to L3-SMI **(D)**. BMI, body mass index.

Poor muscle quality defined according to L3-SMD was significantly associated with shorter OS and PFS (HR = 6.29; 95% CI = 2.17–18.26; *p* < 0.001, and HR = 2.42; 95% CI = 1.26–4.65; *p* = 0.008, respectively). When defined according to L3-SMI, decreased muscle mass was not associated with OS and PFS (HR = 0.70; 95% CI = 0.26–1.85; *p* = 0.469, and HR = 1.47; 95% CI = 0.77–2.82; *p* = 0.248) (Figure 2). No association with OS and PFS was observed for L3-SMI-2 and L3-SMI-3 (Supplementary Figure 2).

Multivariate Cox proportional hazard analyses, with BMI, male sex, IPI, and R-CHOP therapy as covariates, confirmed the lack of significant association of L3-SMI-defined muscle mass depletion with OS and PFS. The association between poor muscle quality defined according to L3-SMD and PFS was not statistically significant (HR = 1.40, 95% CI = 0.69–2.86, *p* = 0.356), while a significant association remained for OS (HR = 3.54; 95% CI = 1.10–11.40; *p* = 0.034) (Table 3).

**Table 3:** Multivariate Cox models for OS and PFS including muscle modification according to SMI or SMD.

	**HR**	**95% CI**	***p***		**HR**	**95% CI**	***p***
**Overall survival**							
L3-SMI-based muscle depletion^a^	0.67	0.25–1.82	0.431	L3-SMD-based poor muscle quality	3.54	1.10–11.40	0.034
BMI	0.98	0.90–1.06	0.603	BMI	1.01	0.92–1.10	0.874
Male sex	1.04	0.47–2.28	0.932	Male sex	1.29	0.57–2.92	0.547
IPI	2.42	1.66–3.53	<0.001	IPI	2.03	1.35–3.04	<0.001
R-CHOP therapy	1.03	0.46–2.27	0.952	R-CHOP therapy	1.12	0.51–2.45	0.785
**Progression free survival**							
L3-SMI-based muscle depletion^a^	1.53	0.78–3.00	0.216	L3-SMD-based poor muscle quality	1.40	0.69–2.86	0.356
BMI	0.98	0.91–1.05	0.509	BMI	0.98	0.91–1.05	0.503
Male sex	1.16	0.62–2.17	0.646	Male sex	1.16	0.62–2.16	0.649
IPI	1.92	1.46–2.53	<0.001	IPI	1.84	1.38–2.46	<0.001
R-CHOP therapy	0.81	0.43–1.51	0.504	R-CHOP therapy	0.90	0.48–1.68	0.737

*Multivariate Cox models for overall and progression-free survival with BMI, male sex, and IPI as covariates. OS, overall survival; PFS, progression-free survival; SMD, skeletal muscle density; SMI, skeletal muscle index; BMI, body mass index; IPI, international prognostic index; L3, third lumbar vertebra*.

a *Results were similar when defining muscle mass depletion according to the other sets of L3-SMI (L3-SMI-2 and L3-SMI-3) cut-off values reported in the section Methods, PET-CT image analysis*.

### Combined Prognostic Effect of Poor Muscle Quality and Obesity on Survival

When stratifying patients according to BMI groups (<25; 25–30; ≥30), no significant difference was observed between groups in terms of OS (*p* = 0.606) and PFS (*p* = 0.437). Similar results were found when stratifying patients according to VAT tertiles (*p* = 0.540 and *p* = 0.549, respectively, for OS and PFS) (Supplementary Figure 3).

**Figure 3:**
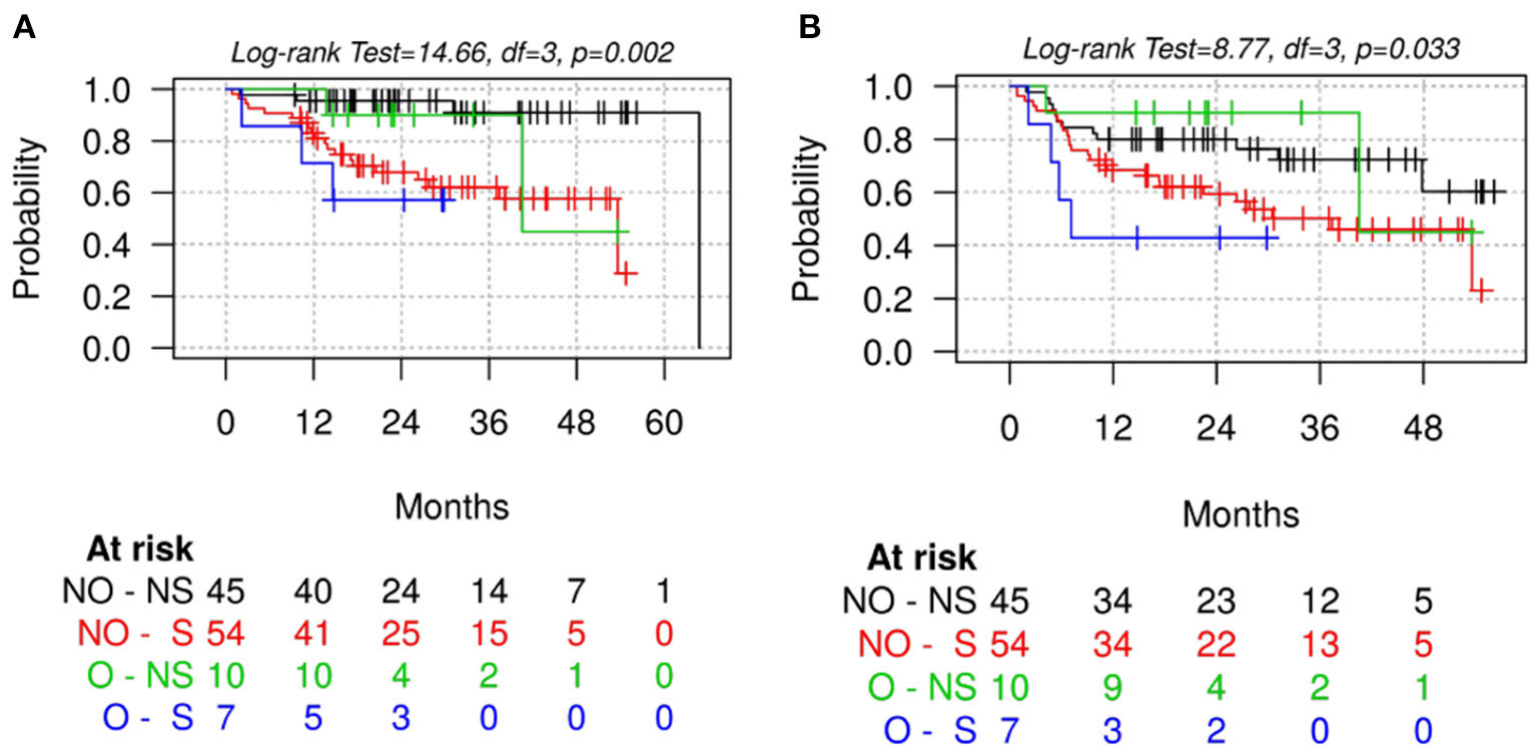
Overall survival **(A)** and progression-free survival **(B)** stratified by poor muscle quality (according to L3-SMD cut-off values) and obesity (defined based on BMI ≥30). S, sarcopenic patients defined according to skeletal muscle density at the level of the third lumbar vertebra (L3-SMD); O, obese patients based on BMI ≥30; NS, non-sarcopenic patients according to L3-SMD; NO, non-obese patients.

When further stratifying patients into four categories according to poor muscle quality defined based on L3-SMD and obesity (BMI ≥ 30), a significant difference was found between categories in terms of OS (*p* = 0.002) and PFS (*p* = 0.033), with obese sarcopenic patients having the worst survival (Figure 3). Similar trends were found when stratifying patients according to poor muscle quality and visceral obesity defined based on VAT or VAT/SAT tertiles, with the worst survivals for sarcopenic patients with higher visceral adiposity (Supplementary Figure 4).

### Impact of PET-CT Indices on Early Therapy Termination

Early therapy termination was experienced by 16 patients (13.8%). In 3 patients, treatment was stopped for lack of response (2 disease progressions, 1 partial response), while in the remaining 13 patients, it was terminated due to grade >2 toxicity (8 infectious complications, 1 deep venous thrombosis, 1 intestinal occlusion, 1 intestinal perforation, 1 neuropathy, 1 cardiac impairment), which led to death in 4 patients.

Poor muscle quality defined according to L3-SMD was significantly associated with early therapy termination: treatment terminations were 13/61 (21.31%) in the poor muscle quality group vs. 3/55 (5.45%) in the group with preserved muscle quality (*p* = 0.028). This association was not found for muscle depletion based on L3-SMI cut-offs (5/29 = 17.2% in patients with muscle depletion, 11/87 = 12.6% in patients without muscle depletion, *p* = 0.542). However, patients who experienced early therapy termination had significantly lower PT-SMI (median: 65.2 vs. 78.2 cm^2^/m^2^, *p* = 0.041) and PT-SMD (41 vs. 48 HU, *p* = 0.023), with borderline significant PT-IMAT (17 vs. 25, *p* = 0.064).

## Discussion

Body composition is known to affect clinical outcome in cancer patients. The assessment of sarcopenia and other body composition indices by means of routinely performed pretreatment diagnostic imaging is of great importance in clinical oncology. We have studied body composition based on DLBCL patients' basal PET-CT images in relation to the clinical outcome. Our findings show that a reduced L3-SMD or PT-SMD or increased PT-IMAT is associated with a low PFS and OS in DLBCL. Poor muscle quality was also associated with early therapy termination. The measurement of sarcopenia indices not only at the L3 level, as explored in the previous literature, but also at the PT level represents a novelty. Considering the absence of cut-off values for PT measurements, these indices were evaluated as continuous variables, and PT-SMD performed as a superior survival prognostic index over L3-SMD. Furthermore, when measured at the PT level, the expansion of intermuscular fat (PT-IMAT) was found to be associated with a significant reduction in OS and PFS. The prognostic value of IMAT has never been described previously in patients with DLBCL.

As a continuous variable, the reduction of PT-SMD showed to be a robust and independent prognostic index of PFS and OS in a multivariate analysis. At the L3 level, if we consider a cut-off of 41 HU for patients with BMI <25 and 33 HU for patients with BMI >25 (10, 29), SMD was an independent negative prognostic factor for OS but not for PFS. The negative correlation between the L3-SMD, serum albumin, and total serum protein values and the positive correlation with PCR suggest an association between muscle fat depots and the systemic inflammatory status of the patients.

The etiology of cancer-associated muscle alterations is multifactorial. A higher IMAT and lower SMD are caused by an increase in inter- and intramuscular fat, respectively, which is often associated with systemic inflammation and metabolic derangement (15). Lymphoma cells or the body's immune response to cancer cells can aggravate the systemic inflammatory response. The inflammatory status and concomitant alterations in mitochondrial function can reduce the ability of the muscle fiber to oxidize lipids, thus increasing fat depots and decreasing the cell energy reserve (14) with the consequence of a reduction in muscle strength and physical performance (36). Besides an ectopic fat deposition, these muscle changes might be indices of a more severe disease or more intense host pro-inflammatory response leading to worse clinical outcomes. We can only speculate why the indices of fat accumulation in the muscles of the PT are stronger predictors than those at the L3 level. A decreased muscle radio-density has been related to reduced strength and performance (37–39), and a reduced quality of the muscles of the lower limb and thigh might be a better predictor of poor muscle function and physical performance status, indicating a more severe sarcopenic status (40, 41). In addition, the muscles of the thigh might be more sensitive to the effects of metabolic or inflammatory derangement. Whatever the cause may be, SMD and IMAT at the thigh level represent new prognostic indices of clinical outcome or predictors of response to therapy in patients with DLBCL.

In our study, a decreased SMD plays a more potent role than obesity, defined by a high BMI, or visceral obesity, defined by increased VAT, in influencing patients' prognosis. However, sarcopenia and obesity can interact and aggravate each other, leading to sarcopenic obesity. The obese sarcopenic patients with a higher BMI had poorer survival outcomes, with similar results when stratifying patients according to VAT or VAT/SAT instead of BMI, suggesting that obesity acts mainly through its visceral component. Thus, our results confirm the combined effect of sarcopenia and visceral obesity previously described in patients undergoing stem cell transplantation (27). However, this combination was never described before in DLBCL patients, especially by using muscle quality rather than quantity to define sarcopenia.

Sarcopenia defined according to a low SMD was also significantly associated with early therapy termination due to toxicity. This result emphasizes the important potential implications that body composition has on the prescription of immunochemotherapy.

We have found that sarcopenia influenced patient survival and early therapy termination only when it was defined as low SMD but not when defined as low SMI, regardless of the use of different cut-offs. This confirms the findings of Chu et al. (6) suggesting that poor muscle quality rather than muscle mass depletion affects survival. In the DLBCL patients that we have investigated, a reduction of SMI had no adverse prognostic value. In fact, in the multivariate analysis, a decreased SMI was associated with significantly improved OS among patients. There is a debate concerning whether sarcopenia due to muscle loss is directly associated with reduced response to anticancer therapy. While a few studies indicated that sarcopenia assessed by a low SMI is predictive of a worse PFS and OS in DLBC patients (5, 7, 8), Chu et al. showed that DLBCL patients with low SMI demonstrated a trend toward improved PFS (6). A possible explanation for the better outcomes in our patients with a low muscle mass and reduced L3-SMI may be related to the exposure to relatively higher plasma concentrations of rituximab due to the lower volumes of distribution and reduced clearance of this anticancer drug (6, 42).

This study has several limitations. First, due to the retrospective nature of the research, prospective confirmation of the results is required. In future investigations, the performance status of the patients should be tested in order to correlate muscle function with muscle fat infiltrations at the thigh level. Second is the therapeutic regimen. While the majority of patients have been treated with R-CHOP, few patients received different therapies. In order to reduce the effect of the therapy variable, the multivariate regression analyses for survival were adjusted for R-CHOP therapy. Finally, we lacked data about weight loss before DLBCL diagnosis, which would have allowed to deepen the knowledge of the nutritional status of the included patients. However, the opportunity to analyze PET-CT scan provided us with a comprehensive assessment of body composition at the moment of diagnosis, including lean mass quality and quantity, and body fat distribution.

In conclusion, we have shown that muscle quality, and particularly the inter- and intramuscular fat infiltration at baseline, at the PT level rather than at the L3 level, are associated with a worse prognosis in patients with DLBCL. A staging PET-CT scan, performed as part of standard clinical practice, may serve as a powerful and inexpensive tool to assess body composition metrics. These indicators can complement classical prognostic indices, such as the IPI, which are based on patient characteristics directly associated with the disease. This information, if confirmed in prospective studies, will pave the way for nutritional and physical activity interventions with the aim of improving the body composition, and accordingly the clinical outcomes, of patients with DLBCL.

## Data Availability Statement

The raw data supporting the conclusions of this article will be made available by the authors, without undue reservation.

## Ethics Statement

The studies involving human participants were reviewed and approved by Comitato Etico dell'Area Vasta Emilia Nord, Policlinico di Modena, Modena, Italy. The patients/participants provided their written informed consent to participate in this study.

## Author Contributions

GB, AV, GL, PP, SC, FMa, SL, FMe, and MP contributed to the study concept and design. GB, FMa, EB, MC, and SV collected the data. GB, FMa, EB, LB, DM, SL, and MP conducted the data analysis and interpreted the results. All authors critically reviewed the final draft of the manuscript and approved the final version of the manuscript to be submitted.

## Conflict of Interest

The authors declare that the research was conducted in the absence of any commercial or financial relationships that could be construed as a potential conflict of interest.
