# Supplementary material for: Prognostic Impact of Muscle Quantity and Quality and Fat Distribution in Diffuse Large B-Cell Lymphoma Patients
Source: Front Nutr. 2021 May 7;8:620696. doi: 10.3389/fnut.2021.620696 (PMC8138563; doi:10.3389/fnut.2021.620696)
Supplement: Supplementary file 1 [file Data_Sheet_1.DOCX]

| **PET-CT indices of body composition** | **All patients (n=116)** |
| --- | --- |
| L3-SMA (cm^2^) | 139.9 ± 34.9 |
| L3-SMI (cm^2^/m^2^) | 50.2 ± 10 |
| L3-SMD (HU) | 34.7 ± 8.1 |
| % of patients with low L3-SMI:   - cut-off values 1 (L3-SMI)^a^ - cut-off values 2 (L3-SMI-2)^b^ - cut-off values 3 (L3-SMI-3)^c^ | 29 (25.0%)  40 (34.5%)  30 (25.9%) |
| % of patients with low L3-SMD | 61 (52.6%) |
| L3-Total adipose tissue area (cm^2^) | 317 (189.5-431.0) |
| L3-SAT (cm^2^) | 140.5 (90-211.3) |
| L3-VAT (cm^2^) | 125 (82.8-207.5) |
| L3- IMAT (cm^2^) | 18.5 (10.0-25.3) |
| VAT/SAT | 0.9 (0.6-1.3) |
| PT-SMI (cm^2^/m^2^) | 78.9 ± 19.1 |
| PT-SMD (HU) | 46.2 ± 6.5 |
| PT-IMAT (cm^2^) | 19 (13-28) |

**Supplementary Table 1**. PET-CT indices of body composition of the patients at the time of diagnosis

Data are reported as frequencies and percentage for categorical variables, means and standard deviations for normally distributed continuous variables and median and interquartile range for non-normally distributed continuous variables. PET-CT, positron emission tomography-computed tomography; L3, third lumbar vertebra; SMA, skeletal muscle area; PT, proximal thigh; SMI, skeletal muscle index; SMD, skeletal muscle density; VAT, visceral adipose tissue; SAT, subcutaneous adipose tissue; IMAT, inter-muscular adipose tissue. For SMI at the level of the third lumbar vertebra, three different sets of cut-off values were used. ^a^L3-SMI: <43 cm^2^/m^2^ for men with BMI <25, <53 cm^2^/m^2^ for men with BMI ≥25, and <41 cm^2^/m^2^ for women; ^b^L3-SMI-2: <55.8 cm^2^/m^2^ for men and <38.9 cm^2^/m^2^ for women; ^c^L3-SMI-3: <52.4 cm^2^/m^2^ for men and <38.5 cm^2^/m^2^ for women.

**Supplementary Table 2.** Correlations between different PET-CT indices of body composition and correlations between PET-CT indices with serological data

| **PET-CT indices of body composition** | **Correlation coefficient (95%CI)** |
| --- | --- |
| L3-SMI and L3-SMD | 0.36 (0.19 – 0.51) |
| L3-SMI and VAT | 0.21 (0.03 – 0.38) |
| L3-SMD and VAT | -0.20 (-0.37 – -0.01) |
| L3-SMI and PT-SMI | 0.65 (0.53 – 0.74) |
| L3-SMD and PT-SMD | 0.86 (0.81 ­– 0.90) |
| L3-IMAT and PT-IMAT | 0.63 (0.51 – 0.73) |
| **Body-composition PET-CT indices and serological data** | |
| Serum Albumin and L3-SMI | 0.20 (0.02 – 0.37) |
| Serum Protein level and L3-SMI | 0.10 (-0.09 – 0.28) |
| C-reactive protein and L3-SMI | -0.12 (-0.30 – 0.07) |
| Serum Glucose and L3-SMI | 0.04 (-0.15 – 0.22) |
| Serum Vitamin D and L3-SMI | -0.04 (-0.25 – 0.17) |
| Serum Albumin and L3-SMD | 0.38 (0.22 – 0.53) |
| Serum Protein level and L3-SMD | 0.41 (0.24 – 0.55) |
| C-reactive protein and L3-SMD | -0.25 (-0.42 – -0.07) |
| Serum Glucose and L3-SMD | -0.07 (-0.25 – 0.11) |
| Serum Vitamin D and L3-SMD | 0.03 (-0.18 – 0.24) |
| Serum Albumin and VAT | -0.00 (-0.18 – 0.18) |
| Serum Protein level and VAT | -0.08 (-0.26 – 0.11) |
| C-reactive protein and VAT | 0.01 (-0.18 – 0.19) |
| Serum Glucose and VAT | 0.35 (0.18 – 0.50) |
| Serum Vitamin D and VAT | -0.05 (-0.26 – 0.16) |

PET-CT, positron emission tomography-computed tomography; CI, confidence interval; L3, third lumbar vertebra; PT, proximal thigh; SMI, skeletal muscle index; SMD, skeletal muscle density; VAT, visceral adipose tissue; IMAT, intramuscular adipose tissue.

A
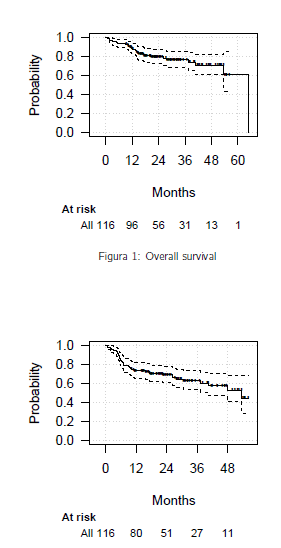
B
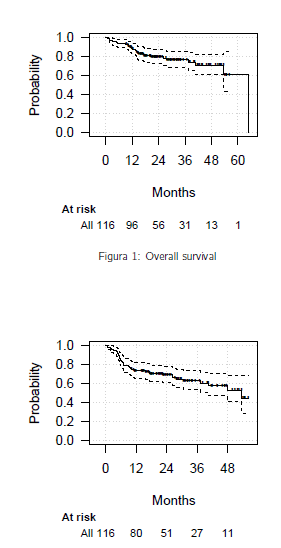


**Supplementary Figure 1.** Overall survival (**A**) and progression-free survival (**B**) with respective 95% confidence intervals in the whole population

A
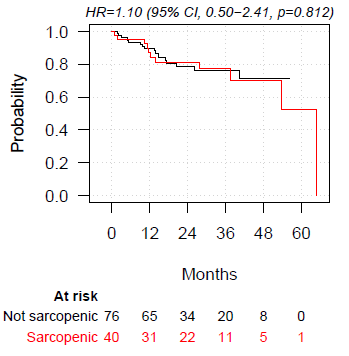
B
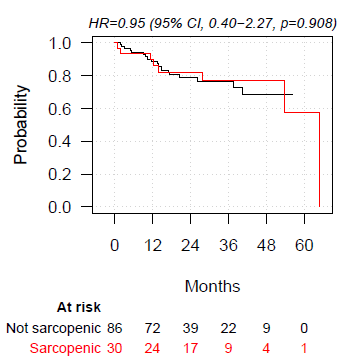


C
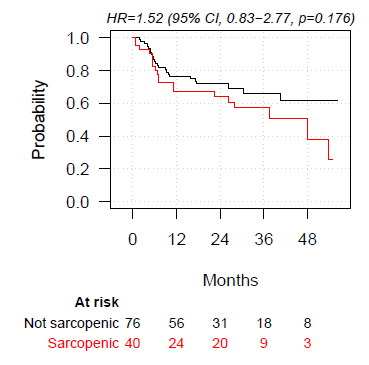
 D
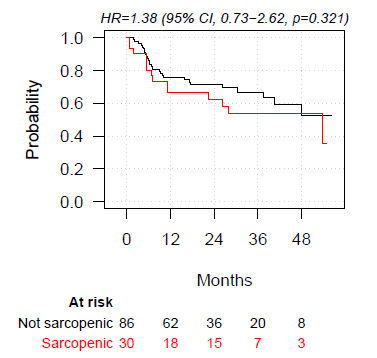


**Supplementary Figure 2.** Overall survival by muscle depletion (sarcopenic) according to L3-SMI-2 cut-off values (<55.8 cm^2^/m^2^ for men and <38.9 cm^2^/m^2^ for women) (**A**), and L3-SMI-3 cut-off values (<52.4 cm^2^/m^2^ for men and <38.5 cm^2^/m^2^ for women) (**B**). Progression-free survival by muscle depletion (sarcopenic) according to L3-SMI-2 cut-off values (<55.8 cm^2^/m^2^ for men and <38.9 cm^2^/m^2^ for women) (**C**), and to L3-SMI-3 cut-off values (<52.4 cm^2^/m^2^ for men and <38.5 cm^2^/m^2^ for women) (**D**). L3, third lumbar vertebra; SMI, skeletal muscle index.

A
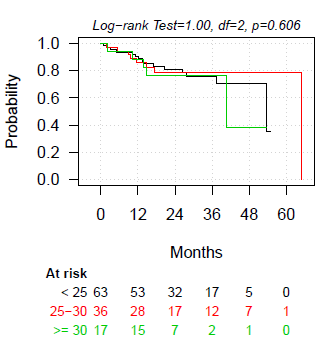
B
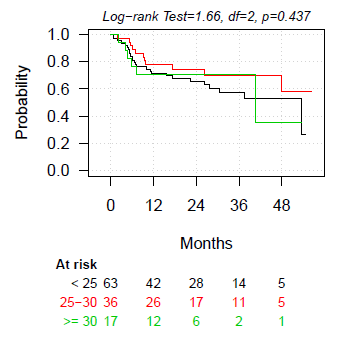


C
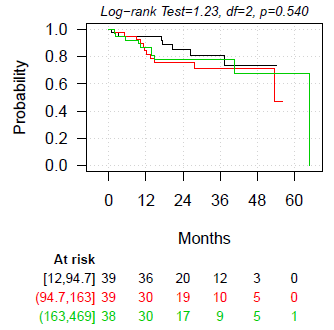
 D
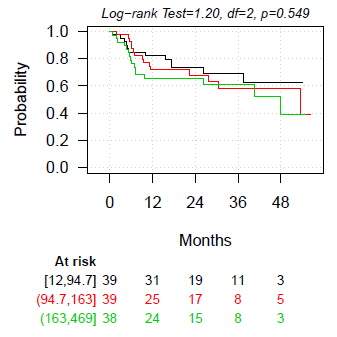


**Supplementary Figure 3.** Overall survival (**A**) and progression-free survival (**B**) by BMI groups (<25; 25-30; ≥30). Overall survival (**C**) and progression-free survival (**D**) by VAT tertiles (12-94.7; 94.7-163; 163-469). BMI, body mass index; VAT, visceral adipose tissue.

A
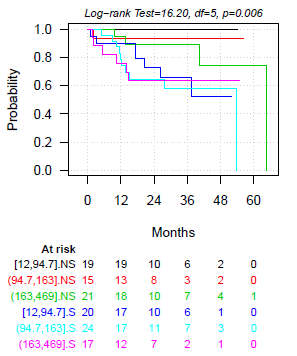
 B
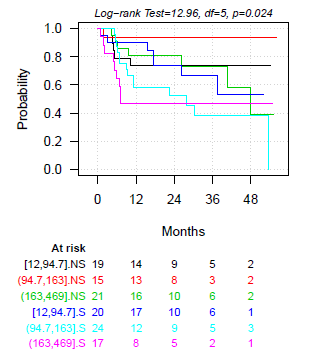


C
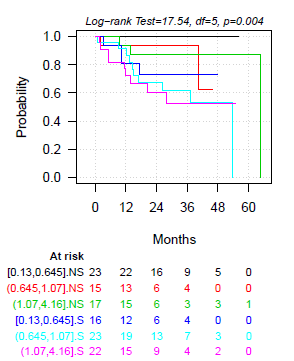
D
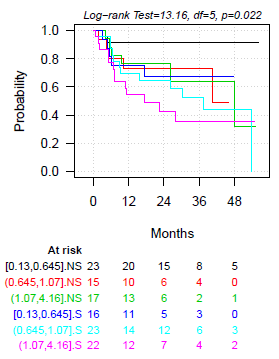


**Supplementary Figure 4.** Overall survival (**A**) and progression-free survival (**B**) stratified by poor muscle quality (according to L3-SMD cut-off values) and visceral obesity (defined based on VAT tertiles). Overall survival (**C**) and progression-free survival (**D**) stratified by poor muscle quality (according to L3-SMD cut-off values) and visceral obesity (defined based on tertiles of VAT/SAT ratio). L3, third lumbar vertebra; SMD, skeletal muscle density; VAT, visceral adipose tissue; SAT, subcutaneous adipose tissue; S, sarcopenic patients defined according to skeletal muscle density at the level of the third lumbar vertebra (L3-SMD); NS, not sarcopenic patients.
